# Effects of dialogic reading for comprehension (LuDiCa) on the social interaction of autistic adolescents and their peers

**DOI:** 10.1186/s41155-023-00283-x

**Published:** 2024-02-02

**Authors:** Victor Loyola de Souza Guevara, Raquel Freire Coêlho, Eileen Pfeiffer Flores

**Affiliations:** 1 Department of Basic Psychological Processes, Institute of Psychology, University of Brasília, ICC SUL–Campus Darcy Ribeiro, Asa Norte, Brasília, Federal District 70910-900 Brazil; 2 Institute of Psychology, Social Work, Health and Human Communication, Federal University of Rio Grande do Sul, Porto Alegre, Brazil

**Keywords:** Dialogic reading for comprehension, Autism spectrum disorder, Adolescence, Social interaction

## Abstract

**Background:**

In today’s contemporary world, relationships take a prominent role in the lives of adolescents. However, challenges related to mutual understanding and a lack of inclusive environments can often lead to autistic teens feeling excluded.

**Objective:**

In order to assess the impact of naturalistic interventions on interpersonal relationships, we conducted an experimental test utilizing Dialogic Reading for Comprehension (LuDiCa) in online reading circles with groups comprising both autistic and neurotypical adolescents. Our focus was on exploring its relevance for enhancing social interaction, particularly in terms of conversational acts, sharing experiences, initiations, and questions.

**Methods:**

Five autistic and five neurotypical students between 11 and 15 years old from a public school in Brasilia, Federal District, participated. We paired groups A and B (made up of trios of teenagers) and groups C and D (made up of pairs) in a multiple baseline design per reading group, in which all groups went through the baseline conditions (BL), intervention (LuDiCa) and maintenance.

**Results:**

LuDiCa increased the frequency of conversational acts of both autistic participants and neurotypical peers. In addition, the intervention favored initiations, questions, and sharing experiences, through the shared activity of reading and talking about a work of fiction. Participants rated the intervention in relation to the platform, the book, the reading facilitator, and interaction with peers. We discuss the potential of the facilitator's role in favoring interactions and the potential of LuDiCa as a joint activity for the engagement of adolescents. We also include suggestions for future research focused on the online context and discuss some limitations of the LuDiCa intervention.

**Conclusion:**

In summary, our study offers initial experimental evidence demonstrating the positive impact of LuDiCa on social interaction behaviors among both autistic and neurotypical adolescents within an inclusive setting.

**Supplementary Information:**

The online version contains supplementary material available at 10.1186/s41155-023-00283-x.

## Introduction

Difficulties in understanding social conventions and engaging in interpersonal communication have been cited as barriers to more fulfilling social interactions between autistic young people^1^ (formally autism spectrum disorder or ASD) and their neurotypical peers, especially in the school environment (Bauminger et al., 2010). Such difficulties are said to be related to some of the diagnostic characteristics, which are said to include difficulties in communication and social interaction with neurotypical pairs (difficulties which are generally perceived as minor or nonexistent in autistic peers, although this is not necessarily true, see Morrison et al., 2020).

The American Psychiatric Association also cites as diagnostic criteria unusual sensory sensitivity (e.g., exacerbated sensitivity to lights or sounds), stereotyped behaviors, and restricted interests (American Psychiatric Association [APA], 2013; Camargo & Bosa, 2009). However, members of the autistic community themselves may often experience so-called stereotyped behaviors as positive and functional. For example, *stimming* (an appropriation and abbreviation of the medical term *self-stimulation*), is seen as having an important function of self-regulation and emotional expression (for example, hand-flapping is often an expression of joy, curiosity or interest) (Ekblad & Pfuhl, 2017). On the other hand, the “restricted interests” pointed out by the diagnostic manual are seen by many in the community not as restricted, but as intense and in-depth. Such interests, which can vary from one period to another or last for many years, are seen by many autistic persons as a strength, not a deficit, as they allow autistic people to devote themselves fully to themes and causes (a recent example is that of Greta Thunberg, who once said she considers her autism a “superpower” that allows her to devote herself intensely to study and activism in favor of slowing climate change (Skafle et al., 2021)). On the other hand, many studies raise the strong influence of prejudices directed at autistic people, especially by potential neurotypical partners, which contribute to relational difficulties (Sasson et al., 2017; Shattuck et al., 2012).

Problems in communication and social interaction that cause suffering in relationships are not only due to adjustments and support needed by autistic individuals but also to the lack of adjustments and barriers in the physical and social environment. Therefore, deficiencies should be seen as the outcome of complex factors, rather than being solely attributed to an individual's personal characteristics. (Hutchison, 1995; Sasson et al., 2017).

Autistic adolescents may have an impoverished social support network and a larger sense of loneliness compared to neurotypical peers and frequently report the desire for more relationships with their peers (Bauminger & Kasari, 2000). In addition, entry into adolescence is marked by a heightened importance given to close and often intense friendships (Buhrmester, 1990). For most adolescents, regardless of neurotype, social relationships occupy a central place, and the way they are accepted by peers is one of the main aspects of their lives (Santrock, 2019). Especially in urban Western societies, the priority given to family bonding and teachers during childhood tends to decrease with the entry of adolescence, and there is often an increase in complexity and a larger appreciation of friendships, intimate relationships and affiliations with restricted groups (known as “cliques”), agglomerations and other peer networks (Brown & Klute, 2003).

Autistic people may find it challenging to maintain and extend topics in conversation with open comments and questions, as well as sustaining reciprocal answers or more than one conversation shift (Jones & Schwartz, 2009; Paul et al., 2009), especially when the topic is not especially interesting to them. In line with the Social Model of Disability^2^, these are considered barriers that become salient when autistic people are expected to follow conventional conversation formats typical of non-autistic people. In fact, there is evidence that conversations between autistic couples often do not present the same obstacles (Morrison et al., 2020).

Carter et al. (2014) have discussed interventions planned for autistic adolescents in the school context, highlighting the potential of this environment for the promotion of social repertoires. The authors remark on the scarcity of interventions tailored specifically to the needs of autistic students in transition to high school. Nevertheless, based on previous literature reviews and their own work in schools, they highlight five guidelines that, in their view, should be considered when planning such interventions: (a) develop social competence in autistic students; (b) improve neurotypical pair interaction skills; (c) improve the support and opportunities offered by teachers; (d) initiate broader efforts across the school community; and (e) involve family members. The authors stress that these general guidelines should be complemented with adjustments for specific individual needs.

There is evidence for the effectiveness of naturalistic interventions for the development of verbal repertoire with autistic participants (Ingersoll & Wainer, 2013; Rao et al., 2008; Rogers & Dawson, 2010; Sandbank et al., 2020; Schreibman et al., 2015). Peer-Mediated Interventions (PMI) are considered naturalistic, since they are implemented by pairs of autistic children, adolescents, or young people, who expand the availability of support and social interaction. In such studies, peers are considered “natural experts” in the art of conversing with people of the same age group, making peer-to-peer interventions a preferred option of intervention among elementary and high school students (Carter et al., 2014).

One example is the study conducted by Bambara et al. (2018), who evaluated the effects of a PMI implemented during high school lunch breaks on the improvement of dialogue skills. The four autistic participants (ages between 14 and 20) were considered by their teachers to be “passive communicators”, that is, young people who responded well to initiations, but rarely initiated conversations themselves, asked open questions, or commented spontaneously. The authors used a single-subject design to evaluate an intervention combining peer-to-peer training and written prompts on conversation skills. Results showed that the intervention improved three conversation skills: (a) mediating interaction through dialogue; (b) initiating; and (c) asking follow-up questions. In addition, the gains extended to conversations with new peers in the last phase of the research. Similar results were reported in other studies using peer-to-peer interventions (e.g., Bambara et al., 2016, 2018; Gardner et al., 2014; Haring & Breen, 1992; MacFarland & Fisher, 2019; Schmidt & Stichter, 2012) for a review, see Guevara, 2021).

Despite the positive results, there are a few important limitations to peer-to-peer interventions as they have been studied so far. First, the frequent absence of evaluations of the intervention by the autistic participants (e.g., MacFarland & Fisher, 2019); (2) the selection of peers by teachers/school staff, instead of considering similar and previously shared interests (Bambara et al., 2018; Gardner et al., 2014; Haring & Breen, 1992; Schmidt & Stichter, 2012); (3) the presence of adults prompting and guiding the interaction (e.g., Haring & Breen, 1992; MacFarland & Fisher, 2019); and (4) the lack of a joint activity that might favor the engagement, as there is evidence that autistic persons tend to prefer dialogue around a shared activity, to simply talking (Bottema-Beutel et al., 2016).

Since verbal behavior is always interaction (Skinner, 1957), it seems incorrect to conceptualize communication difficulties as being inherent to individuals, or even traceable to “deficits” attributable to only part of the interaction. Verbal interaction always depends on both parts and any problem in this interaction can be interpreted by looking at how the parts influence each other. In the PMI studies, however, the methodology implicitly assumed that it is possible to improve interaction by focusing the intervention on the behavior of one of the parties, in this case, always the autistic party.

Adopting the principle of charity, we might presume that the idea behind current PMI studies is that helping one part of the interaction will start a virtuous cycle of better communication. However, this does not justify why the focus would always be on the autistic party. Furthermore, Skinner’s Verbal Behavior invites us to conceptualize verbal behavior as necessarily interactive, in which the refinement is mediated by the listener’s behavior and, crucially, the roles of listener and speaker alternate (Skinner, 1957). Therefore, so-called communication problems cannot be attributed to only a portion of the interaction. Consistency with the functional approach calls for broadening our gaze and being consistent with the basic principle that *any* interaction cannot, *by definition,* be attributed to inherent characteristics, but only to socially shared contingencies, which, as den Houting (2019) points out, are largely physical, socially and emotionally non-inclusive for autistic individuals.

Aiming to contribute to current investigations on the benefits of PMI, whilst also seeking to overcome said limitations, we planned an experimental test of a situation in which peer dialogue would occur around a shared reading activity, using the LuDiCa methodology 3 (for an explanation of the rationale behind LuDiCa, see Flores et al., 2020, b; for an example of application, see Moraes & Flores, 2020). The idea behind this choice is that common topics of conversation would arise more naturally around this shared activity, rather than, as in previous studies, assuming the “conversational expertise” of neurotypical peers or imposing conversation topics and prompts.

Dialogical reading for comprehension (LuDiCa) is a shared-reading intervention in which the facilitator dialogue, especially through open questions and acknowlodgments (e.g., paraphrasing them, praising them, or expanding them) (Moraes & Flores, 2020). LuDiCa’s differential in comparison to other forms of dialogic reading is that invitations to dialogue and feedback are based on careful prior analysis of narrative events and functions (for an explanation of these concepts, see Flores et al. (2020, b), and also the “Method” section). Recent studies suggest that the LuDiCa method benefits reading comprehension in children (Flores et al., 2014; Medeiros & Flores, 2016; Rogoski et al., 2015) and adults (Moraes & Flores, 2020). There is also evidence that, when used with autistic children, it can help foster joint attention (Caldas & Flores, 2020), as well as engagement and language (Guevara et al., 2017; Queiroz et al., 2020).

LuDiCa happens in a series of repeated “dialogical cycles” (for a detailed description, see Bisello, 2018) as follows: During shared reading, the facilitator stops at crucial points (defined by the presence of an important narrative function and by a “natural” pausing point) and invites participants to join in conversation about and around the story. The facilitator also helps the flow by maintaining a responsive attitude (e.g., asking follow-up questions and commenting) and by encouraging turn-taking and participation of all involved.

As we have seen, most studies with PMI have considered it as given that neurotypical adolescents are “natural experts” (Guevara, 2021). Recent studies, however, have pointed out that difficulties in communication are caused by the neurotypical person’s inability to understand the autistic person’s communicative acts, as much as the opposite. In other words, it involves what Damien Milton (2012) coined as the “double problem of empathy”. We thus propose that PMI should not assume that neurotypical peers are experts in communication. In our intervention, using LuDiCa, we will measure not only gains in autistic participants’ communicative skills but in all of those involved.

Another difference in the intervention we propose, compared to previous studies using PMI, is that we use a common activity (shared reading). Listening and discussing a story may have potential advantages compared to (a) general prompts (e.g., “get to know each other”) or (b) very specific prompts (such as talking about dinosaurs or television series). The LuDiCa situation naturally offers various possible topics for discussion, about and around the story. At the same time, it also makes it less awkward to remain in silence, if one so wishes.

### Objective

Our objective in the present study was to verify the effects of LuDiCa in groups with autistic and neurotypical adolescents, on measures of participation and interaction, at the individual and group levels. Our research questions were: to what extent does LuDiCa influence conversational acts, initiation, acts of metacommunication, questions, and the sharing of personal experiences? What discernible disparities exist in the participation of neurotypical and neurodivergent adolescents within the inclusive dialogic reading context? How do adolescents assess their engagement in inclusive dialogic reading within an online context?

## Method

Due to the transmission characteristics of COVID-19 and the prevention guidelines held forth by Brazilian health authorities during the data collection period (between April and August 2020), we carried out all stages of the study remotely. Social interactions among the adolescents occurred exclusively through video calls with access to video and audio by all participants. We considered the possible impacts caused by the long period of restriction and changes imposed on schools on the mental health of adolescents (Araújo et al., 2020).

### Participants

Participants were five autistic students and five neurotypical students, aged 11 to 15 years, from a public elementary school (fifth to ninth grade) located in Brasília, Federal District. The selection criteria encompassed consistent attendance of the students at school, diagnosis of autism for autistic participants (presented by the pedagogical team or by family members), explicit interest in participating, and access to a device with a reliable internet connection (e.g., computer, tablet, smartphone) during scheduled times.

Before starting the sessions, participants completed the Aspie Quiz (Ekblad, 2013) (translated version for Brazilian Portuguese available for free at https://rdos.net/eng/Aspie-quiz.php). The test was not used to establish clinical diagnosis (all autistic participants already had a formal diagnosis of Autism Spectrum Disorder) but rather to verify participants ‘perception of neurodivergent characteristics in themselves. This tool is supported by evidence (Ekblad, 2013; Ekblad & Oviedo, 2017), and values the self-identification of each participant with neurodivergent and/or neurotypical characteristics. The result is identified with two different scores: Aspie score (based on the primary factor—neurodiversity) and neurotypical (non-autistic) scores. The test considers results of “very likely Aspie” (neurodivergent) if the Aspie score is at least 35 points higher than its neurotypical score, and “most likely neurotypical” if the neurotypical score is at least 35 points higher than the Aspie score. The intermediate interval is judged as “Aspie and neurotypical traits” (mixed).

The results of the Aspie Quiz can be seen in Table 1, as well as participants’ pseudonyms, their ages and school years, and their prior experiences with shared reading and video conferencing applications.

**Table 1:** Participants’ data and group division

Participants	Age	Grade/School year	Aspie Score (Aspie Quiz)	Neurotypical Score (Aspie Quiz)	Prior experiences with shared reading		Prior experiences with video conference applications
					Virtual	In person	
*Group A*							
Arnaldo (nd)	15	8°	87	107		X	Almost never
Antônio (nt)	14	8°	56	145		X	Occasionally
Ana (nt)	14	9°	109	113		X	Almost never
*Group B*							
Bernardo (nd)	12	7°	140	56	X	X	Occasionally
Bento (nd)	11	6°	113	82			Occasionally
Benjamim (nt)	12	6°	100	126			Daily
*Group C*							
Carlos (nd)	14	7°	97	94		X	Almost never
Cláudio (nt)	12	7°	46	129			Occasionally
*Group D*							
Daniel (nd)	14	7°	75	121			Almost never
Débora (nt)	13	7°	27	193			Daily

All fictitious names were created with the initial letter referring to the group

*nd* neurodivergent, *nt* neurotypical

Table 1 also shows the reading group to which each participant was allotted (randomness was limited by the availability of schedules of each and the criterion of having autistic and neurotypical participants in each reading group). Groups A and B were left with three students in each and groups C and D with two students.

Parents or carers of autistic students were interviewed prior to the experiment. The interview was created by the authors with the objective of surveying the social interactions of adolescents in contexts outside of school, as perceived by their parents. We opted not to employ an established protocol for evaluating the social interactions of adolescents rooted in our intention to refrain from passing judgment on or attempting to alter the “social skills” of autistic individuals. The interview surveyed aspects of social awareness; social cognition; social communication; social motivation; and restricted interests and repetitive behaviors (Additional file 1). For each category, parents answered how they believed their children would interact in hypothetical social situations. The findings of the interviews served as supplementary qualitative insights into the social interactions of autistic students. This enriched dataset was instrumental in enhancing and contextualizing the social validity data for comparative analysis.

### Selection and training of the LuDiCa facilitator

We selected the facilitator for her experience working with LuDiCa (approximately 10 years). She is a psychologist with clinical experience, and a former facilitator at the outreach project *Livros Abertos* (University of Brasilia)*,* which uses the LuDiCa methodology (for a description of the project, see Moraes & Flores, 2020). The first author and facilitator held two training sessions lasting 60 min each before the beginning of data collection, as well as short feedback sessions after each session of data collection.

### Planning of dialogue prompts, based on narrative functions and narrative events

For baseline and intervention sessions, we used the youth novel *A Bolsa Amarela* (The Yellow Bag), by the Brazilian writer Lygia Bojunga (Bojunga, 2006). It tells the story of Raquel, a 10-year-old girl who carries in her yellow bag three secret wishes: to be grown-up, to be a boy, and to become a writer. The work mixes realism (e.g., the difficulties in being a girl and the youngest among much older siblings, the existential anxieties of growth, the frequent contempt of adults for the opinion of young people) with the dimension of the fantastic (e.g., the magic yellow bag). The book is considered a classic of youth literature and the author has received worldwide recognition.^4^

The facilitator's interventions were performed based on a previous analysis of the narrative into events and narrative functions (Flores et al., 2020b). A narrative function is a meaningful functional unit, which is not itself an event, although it is often expressed by various events which happen throughout the story. It is, in other words, what the events “show” or “express” (for example, “Raquel dreams of becoming a writer”. The events are what happens, chronologically, in the story, for example, (1) Raquel wakes up in the middle of the night; (2) she realizes the talking rooster Terrible, is missing from her magic bag; (3) she finds a note left by Terrible. Neither events nor functions are taken directly from literal chunks of the text but rather are listed based on an analysis and summary of these two narrative dimensions.

The concepts of function and event (see Flores et al., 2020b) were developed based on the narratology of Roland Barthes (Barthes & Duisit, 1975) and Skinner’s notion of thematic units (Skinner, 1957). Once the text is analyzed, the resulting events and functions are used as the ground for planning invitations to dialogue during shared reading. The method has been used in recent studies which employed the LuDiCa methodology (e.g., Flores et al., 2014; Medeiros & Flores, 2016; Moraes & Flores, 2020) and is explained in detail in Flores et al. (2020a), Flores et al. (2020b).

The excerpts were analyzed into events and functions by the first author, and a random sample of 27.55% of these excerpts was then also analyzed by the facilitator. Agreements ranged from 97 to 100%.

For each excerpt to be read in each session, we established where in the text the facilitator would prompt dialogue. These invitations were defined to occur at three points in the story, based on three narrative functions detected in our previous analysis (see model of analysis of functions and events in Additional file 2). Invitations were always given through open questions (e.g., how do you think Raquel felt when… happened?). There were also planned pauses for soundcheck, potential difficulties with virtual communications, etc.

### Experimental design and procedure

We used an adapted multiple Baseline design by reading group, in which four reading groups passed through baseline (BL), intervention, and maintenance conditions. In order to avoid excessively long Baselines, we paired Groups A and B and Groups C and D for staggering purposes.

The reading group sessions took place twice weekly. From April to June 2020, sessions took place during what would usually be school time (schools had been temporarily closed due to COVID-19) and then, from June to August, during school time, using free time allowed by their teachers. All sessions were conducted online using Cisco Webex Meetings (Cisco Systems, 2020) and Zoom (Zoom Video Communications, 2020). All sessions were recorded using the applications. The first author, the facilitator, and participants of one of the four groups would be present at each session.

#### Preparatory meetings

Before collecting data, we conducted a 60-min video call with each group to introduce the research, explain participation rules, and familiarize the students with the experimental environment (i.e., video conferencing app layout). We emphasized the items in the Informed Consent Term and Commitment to Data Confidentiality, which each participant signed remotely. The term outlined the stages of the research, participants’ rights, and responsibilities for the collected information, specifically emphasizing the prohibition of using the contents of the meetings for other purposes.

Given the unique nature of video call data collection and to promote interaction, we established guidelines for participating in online reading groups: (a) remain attentive during the video call, avoiding other activities; (b) choose a location with internet access and privacy, using headphones and ensuring the device is charged; (c) arrive on time for the meetings, respecting other members; (d) keep cameras and audio on; (e) feel comfortable commenting and asking questions about the story; (f) enjoy the story, engage with others and interact during the reading group.

During the meeting, the facilitator led the reading of a short story, taking breaks to encourage dialogue and promote participation among the adolescents. This helped to build a connection and familiarize them with the context of the reading group and the video calling platform. The short story read was “The Old Woman of the Forest” from the book “Wonderful Children’s and Household Tales of the Brothers Grimm” (Grimm and Grimm, 2012).

#### Baseline

During baseline (BL) conditions, the groups of adolescents participated in simple shared reading sessions. The facilitator, as in all conditions, read the text expressively and theatrically, and participations were acknowledged. However, the facilitator did not initiate further discussion or make additional interventions.

The sessions were structured around three main moments, led by the facilitator: (1) Opening: the facilitator resumed the point in the story where the group had left off in the previous session, asking questions such as “Guys, do you remember where we stopped? What do you remember?” On the first session of BL, instead of resuming the story, the facilitator asked about the cover, title and author of the literary work: “What do you think this story is about? What do you see on the cover?” (2) Simple shared reading of an excerpt from the book, with at least three follow-up breaks during which the facilitator would ask “All right so far, folks? Do you hear me right? Any questions or comments?” (3) Wrap-Up: The facilitator would ask “ What were your thoughts on the excerpt? Did anything else catch your attention or stand out for you?"

#### Intervention (LuDiCa)

**Fig. 1:**
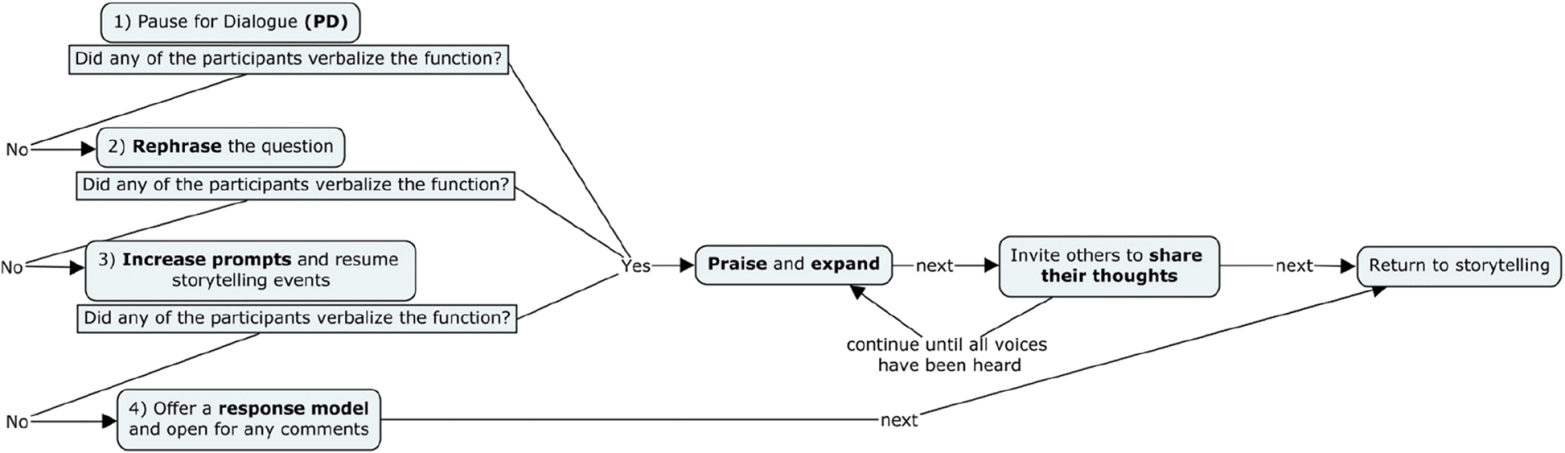
Flow of facilitation strategies on LuDiCa

In the intervention phase of the study, we implemented the LuDiCa methodology. The basic procedure was the same as in the Baseline condition, but additional pauses for dialogue were incorporated according to the LuDiCa methodology. Each reading group session consisted of three pre-established Pauses for Dialogue (PD) led by the facilitator. The facilitator used two types of questions at each pause: (1) questions to verify understanding and encourage engagement with the story, based on narrative functions; and (2) distancing questions, which invited the adolescents to share personal experiences related to the story. For each question, the facilitator followed the procedure illustrated in Fig. 1.

This procedure began with less explicit prompts and progressed to more explicit prompts as needed, using a least-to-most hierarchy, which has been shown to be effective in research with autistic individuals (Schnell et al., 2020). Figure 1 represents the flow of facilitation strategies used during PD. The flow is an adaptation based on the works of Medeiros and Flores (2016) and Moraes and Flores (2020).

#### Maintenance

In the maintenance (Mn) condition, the reading sessions were similar to the BL condition, but different stories were used. We read three tales (one for each session) from the book “Wonderful Children’s and Household Tales of the Brothers Grimm” (Grimm and Grimm, 2012).” The Three Brothers,” “The Doctor Knows-It-All,” and “The Lion and the Frog.”

#### Observational scheme (dependent variables)

The behavioral categories used to analyze interactions were based on the theoretical framework laid out in our introduction with the two major assumptions that (1) interactions are a two-way process and (2) both neurotypical and neurodivergent participants are learning to communicate and interact. The categories of conversational acts, questions, and sharing (defined below) seek to capture, together, dimensions of engagement and interaction, interest in what others have to say, and comfort in speaking about oneself.

**Fig. 2:**
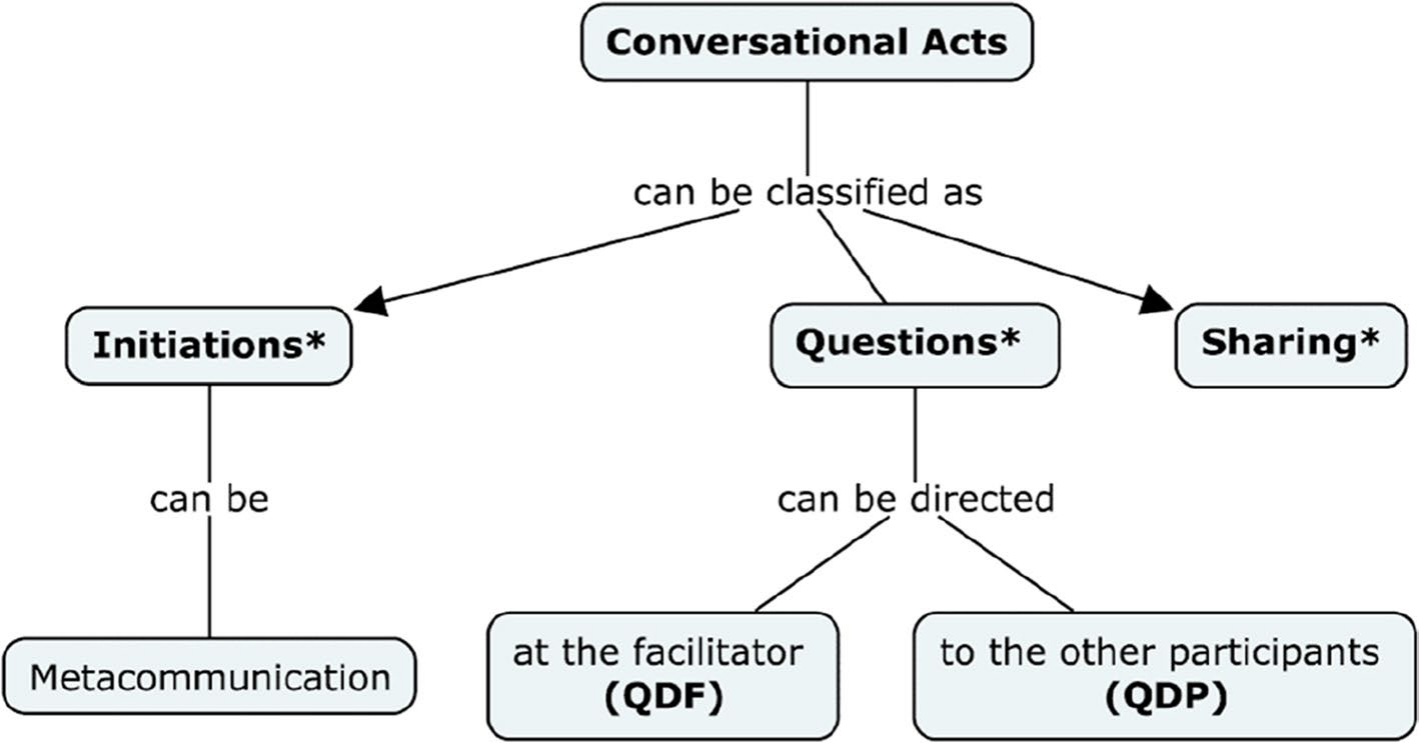
Interaction observations

In order to evaluate how LuDiCa favored social interaction for autistic and neurotypical adolescents, we observed the frequency of conversational acts, including initiations (with a subcategory of metacommunication), questions, and sharing. These observations were recorded and are represented in Fig. 2.

A conversational act is defined as a turn taken during a conversation between participants and/or facilitator. For example, in the scenario provided, the facilitator’s question, “Why do you think the rooster would like to change its name?” would be considered a conversational act Then, P1’s contribution “I don’t know... I think the rooster was tired of being mistaken for a “king” or someone powerful” would be considered one conversational act, and P2’s response “It’s... He wanted to be seen as someone simpler, so King certainly wasn't the most appropriate name for the rooster, was it?” would be considered another conversational act.

Among the conversational acts, we recorded three subcategories to help us gauge engagement, interaction, and interest: (a) initiations—defined as any conversational acts about or around the story that did not happen as a response to a request or direct question from the facilitator (in other words, participation in the conversation that was not directly prompted by the facilitator); (b) metacommunication, in which the participants’ statements were related to the online context, quality of the transmission of information or comprehension of the story. (e.g., “Guys! Can you repeat the part after Raquel met Terrible?” (c) Questions—when participants formulated questions about or around the story**,** directed at the facilitator (QDF) or to the other peer (QDP); and (d) sharing—when participants would express a personal experience, feeling, or interpretation (e.g., “Wow, it made me sad to realize that she was left all alone”; “I would feel very happy if my friends did this for me”; “Something like that happened to me when I changed schools”; “I think she must be sad, but if it were me... I would be angry. I'm a very... I get annoyed easy, you know?”). The categories of interaction, such as initiations and questions were not mutually exclusive, as illustrated in Fig. 2, because we sought to capture all initiations first, as a measure of the active role of participants and inside initiations, to specifically capture questions, which are special in this context in that they show interest in what others have to say.

#### Data processing and analysis

**Fig. 3:**
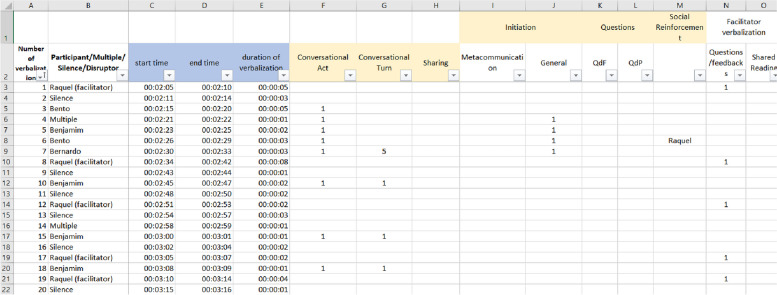
Exemplification of the base for data analysis

First, all videos were analyzed into what we called the Base. This consisted of registering, on a spreadsheet, the beginning and end time of every utterance, so that each count of a behavioral measure could be traced back to a particular time and utterance in the video (see Fig. 3).

Among the utterances, those eventually addressed to other people in each participant’s non-virtual environment were not included in the analysis (e.g., to sibling “Get out of here, I’m busy!”). Moreover, due to practical limitations, we did not analyse most nonverbal interactions, with some exceptions (see below). Initial rapport and housekeeping interactions (e.g., “Let’s see if P is trying to get in”) were also excluded from the analysis.

Besides utterances, other events registered on the base were (1) quick agreements—signs of agreement or interest (*uhum*, *yes, that's right.)* that happened at the same time as someone else’s utterance; (2) multiples: utterances in which two or more participants spoke at the same time and separate utterances could not be discriminated; (3) silence—two or more seconds time with no utterances and (4) disruptors—noises or interruptions from participants ‘homes, interferences, etc.

#### Intervention fidelity

We elaborated two protocols for evaluation of treatment fidelity, separately for the phases of (1) baseline and maintenance; and (2) LuDiCa (Additional file 3). The first protocol checked whether the facilitator, according to the BL and Mn condition, was responsive to possible initiations of the adolescents, but without following and without initiating dialogues. The second protocol checked for the correct application of the LuDiCa flow (Fig. 1). Both protocols also checked for procedures during opening and wrap-up.

We analyzed treatment fidelity in 33.33% of the sessions performed (six baseline sessions, four maintenance sessions, and 26 intervention sessions) randomly selected from each of the four groups. The facilitator proceeded according to protocol in 100% of the sessions.

#### Inter-rater reliability

We tested inter-rater reliability of behavioral measures by analyzing a random sample of 25.92% of sessions, for a total of 28 sessions, including all experimental groups and conditions. The first author a graduate student in Behavioral Sciences with experience in applied research analyzed the equivalence of measures in each session. The agreement was calculated based on the formula (total agreements found/ [agreements + disagreements] × 100) for all measures, with a minimum target of 80% agreement. In cases where agreements were below this rate, the authors reviewed the videos and discussed disagreements. The mean and interval of the agreement found in the first round were: conversational acts (99.61%; 90–100%), sharing (54%; 0–100%), metacommunication (42%; 0–100%), initiation (49%; 0–100%), questions directed at facilitator (QDF) (52%; 0–100%), and questions directed at peers (QDP) (67%; 0–100%). After the discussion, improvement of definitions, and new independent reanalysis, the agreement rate improved to: sharing (99.83; 97–100%), Metacommunication (100%; 100–100%), initiation (100%; 100–100%), QDF (100%; 100–100%), and QDP (100%; 100–100%). It is important to emphasize that the ratings were not simply “yes” or “no”, but rather a classification of each unit into one of the six categories. Chances of agreement by chance were significantly minimized, justifying the use of percentage agreement as a satisfactory indicator of the robustness of our categories.

#### Social validity

We evaluated the opinions of all participants, both autistic and neurotypical, about the use of video call applications using a Likert scale and open-ended questions sent via WhatsApp (WhatsApp LLC, 2020). Participants were able to respond by text or audio. They were asked about the quality of the chosen literary works, their personal performance in relation to the other members of the group and the facilitator, as well as any suggestions or concerns they may have.

The facilitator was also interviewed about her participation, the quality of the relationship with adolescents, and the impact of participating in this study. We also collected comments from the autistic adolescents’ family members throughout the data collection.

## Results

### Effect size

**Fig. 4:**
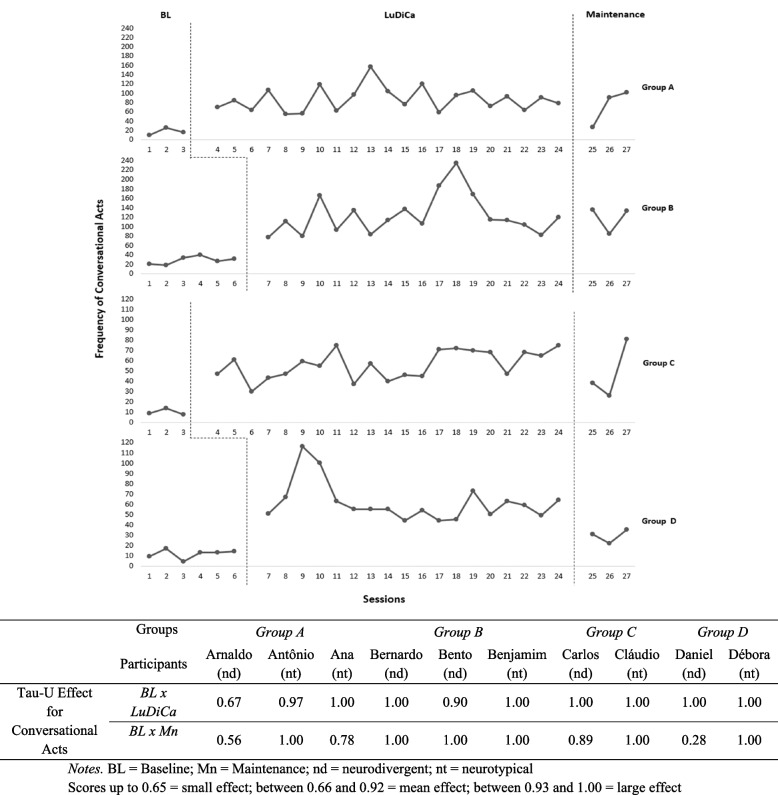
Frequency of conversational acts and Tau-U effect size of LuDiCa per participant

**Fig. 5:**
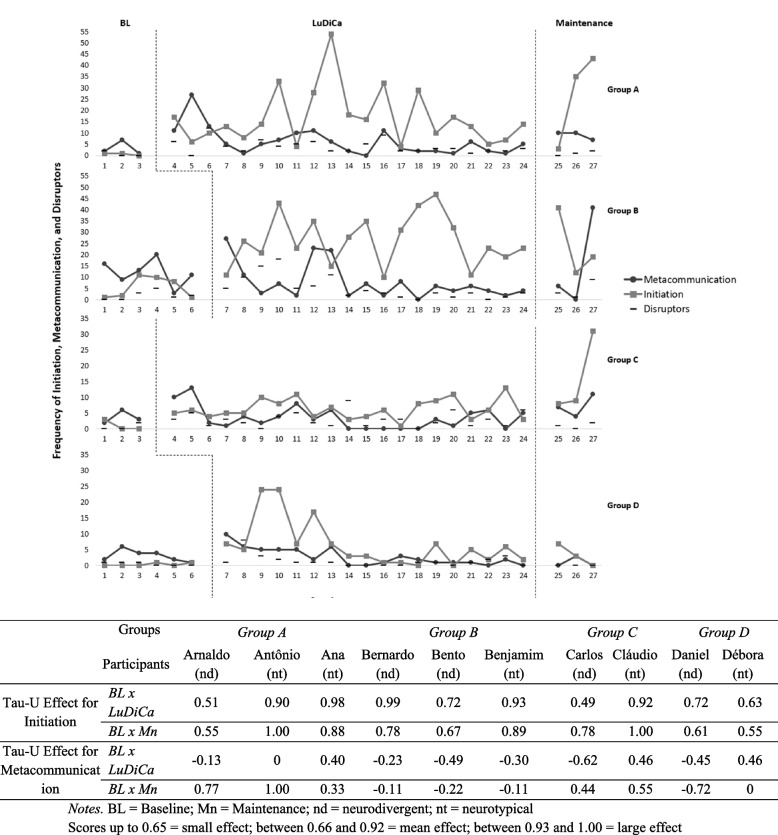
Frequency of initiation and metacommunication and Tau-U effect size of LuDiCa per participant

**Fig. 6:**
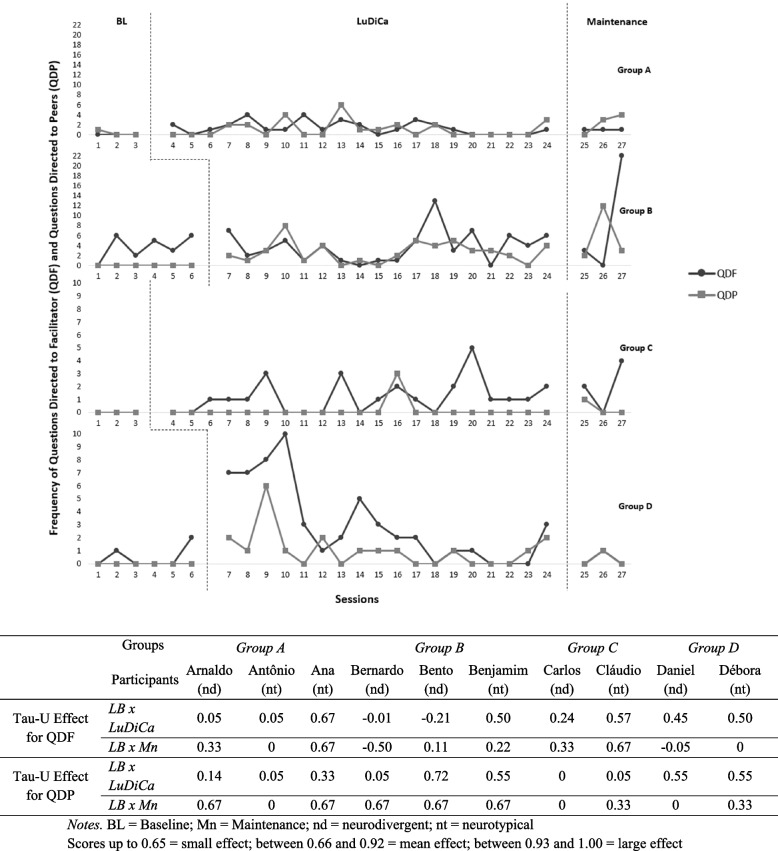
Frequency of QDF and QDP and tau-U effect size of LuDiCa per participant

**Fig. 7:**
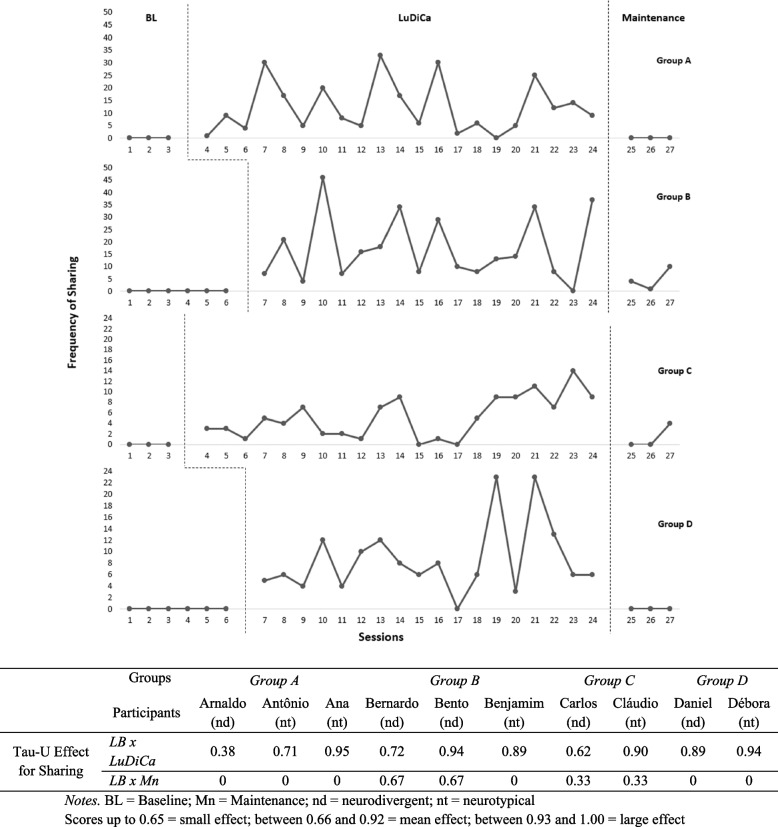
Frequency of sharing and Tau-U effect size of LuDiCa per participant

We used the Tau-U AxB (Vannest et al., 2016) to check for effect sizes by comparing BL with LuDiCa and BL with Maintenance (Mn) for all measures (Figs. 4, 5, 6, and 7).

### Conversational acts

In all groups, both autistic and neurotypical participants increased the frequency of conversational acts with the introduction of LuDiCa, as shown in Fig. 4. The increase was immediate and contingent on the introduction of the LuDiCa methodology. In addition, we noticed stability of the effects of LuDiCa, albeit with larger variability in group B throughout this phase. During Mn, apart from group D and Session 25 of group A, all groups showed rates of conversational acts close to those presented during LuDiCa.

As shown in Fig. 4, in group A, there was a large effect of LuDiCa on the conversational acts for Ana and Antônio (nt), and a mean effect for Arnaldo (autistic). In Group B, the effect of LuDiCa was large for Bernardo (autistic) and Benjamin (nt), and medium for Bento (autistic). In groups C and D, there was a large effect of LuDiCa for all participants.

The large effect on conversational acts continued during the maintenance phase for Antônio, in group A; for all of group B; and for Cláudio and Débora (nt), respectively from groups C and D. There was a medium effect for Ana and Carlos, and a small effect for Arnaldo and Daniel (autistic).

### Initiations and acts of metacommunication

Figure 5 shows initiations (conversational acts that do not occur immediately after and in response to a prompt from the facilitator). The figure also shows instances of metacommunication—conversational acts of the participants aimed at checking or guaranteeing the quality of communication.

Initiations increased with the introduction of LuDiCa for all groups and the pattern persisted during Mn for all groups, except for sessions 25 in group A and session and 27 in group D.

We noticed that this group effect was identified for both neurotypical adolescents and autistic adolescents. There was a large or medium effect of the intervention on initiations for all autistic participants, except for two (Arnaldo and Carlos), and for all neurotypical adolescents, except one (Deborah). However, the large effect was not sustained during Mn.

Also in Fig. 5, we see that, while initiations related to the story were contingent on the introduction of LuDiCa, metacommunication tended to occur across all conditions and to increase in sessions with more disruptors (e.g., sessions 11, 12, and 16 in group A; 4, 12, 13 and 27 in group B; 5 and 11 in group C).

### Questions formulated by participants

Questions formulated by participants about or around the story helped us assess interest and engagement and were divided into those directed to the facilitator (QDF) or to peers (QDP). Figure 6 shows that except for group B, questions directed to the facilitator were rare or absent during BL and appeared or increased slightly during LuDiCa. The effect was small for all participants, except for Ana (nt) (average effect).

Peer-to-peer questions were also absent or rare during BL. With the introduction of LuDiCa, participants in groups A, B, and D began asking each other questions. In group C, the questions were always addressed to the facilitator. The pattern established during LuDiCa persisted throughout the Mn session for all groups.

### Sharing

Figure 7 shows how often participants shared their own experiences and perspectives. There were no instances of sharing during BL, even though, as we explained, pauses for dialogues were included throughout the conditions. Sharing began to happen after the introduction of the LuDiCa methodology, for all groups.

The Tau-U indexes also show that this increase in sharing with the introduction of LuDiCa was large or medium for all autistic participants but one (Bento), and for all neurotypical adolescents. Sharing, however, was, in general, not preserved during Mn, having returned to Baseline levels for groups A, C, and D.

### Social validity

#### Video calling platforms, story and facilitator

All participants except one (Daniel) rated the use of the video calling platform as either excellent or good. Bernardo complained about the delay in the rhythm of interaction when turning microphones on or off, and Deborah suggested that platforms requiring less memory should be used in future studies.

The book (The Yellow Bag) was considered excellent or good by all participants. In the initial sessions, Bento expressed annoyance at the main character as “boring”, “childish” and “a complainer”, but as the story progressed, he commented that, “in fact, her family is the one that’s boring!”, which suggests an increase in his empathy with the protagonist of the story. Ana commented that she wished the story could last longer. The facilitator’s participation was considered excellent or good by all participants, with three adding explicit expressions of appreciation: “Excellent” (Bento), “the facilitator was really nice” (Daniel), and ‘the facilitator did an excellent job” (Ana).

#### Relationship between peers, self-assessment, and learning

Six participants evaluated the relationship with other members of their group as “excellent” or “good to excellent”. And two participants as “good” (Ana and Bento). Daniel suggested having more boys in the group.

Assessment of their own participation was rated as “excellent” by three adolescents (Cláudio, Débora, and Daniel), “good to excellent” by three others (Bernardo, Benjamin, and Carlos) and “good” by four adolescents (Ana, Antônio, Arnaldo and Bento). Bento considered the interaction as, at times, boring. As for what they learned from the experience, Arnaldo stated that he “learned to listen more”, and that “hearing stories can be something cool”. Antônio said that he “learned to share opinions”. Ana commented that the participants in her group learned to get along better with time and added that the experience of “being able to express themselves freely” was an opportunity to “learn and reflect on their personal lives”.

Bento asserted that he “learned to have more patience”; Bernardo said that his interactions were almost non-existent at first but improved throughout the sessions; Benjamin expressed that members in his group learned to “help each other in in bad times” and that he believes that the project “could change the lives of many children and adolescents”. Carlos commented that he “learned to talk about the story and to listen to the facilitator; Claudio said he “learned a lot and found the meetings very cool”. Daniel stated that he “learned that it is possible to tell stories and interact with new people” and considered the experience “Dope” and Deborah said she’d love to hear more stories and that the book was really good. She also mentioned having often identified with the main character.

#### Criticisms and suggestions for change

As for the less appreciated aspects, Bernardo expressed concerns about the absence of active participation by the first author, who was present during sessions but only talked during Opening and Wrap-Up. Carlos cited the eventual low quality of the video, and Bento, the excessively long duration of some sessions. Daniel suggested that conversation moments could include other topics^5^ and that there could be more participants in the groups.

#### Family members

Family members cited the initial resistance of some participants to the virtual format, but also the growing familiarity and autonomy in their use of the platforms. Also mentioned was a growing tendency for members to turn to their peers for help (e.g., one participant, who initially used their mother’s e-mail account, was helped by the others in the group in setting up his own account). Several family members declared being pleasantly surprised by their children’s motivation to participate, expressed by punctuality and requests to family members to avoid interrupting them during sessions. They were also pleased by how much the adolescents understood and engaged with the story, sometimes after initial worries that their children might struggle to follow the story or the discussions.

#### Facilitator

The facilitator reported having enjoyed the story and the interactions throughout the study. She stressed the importance of the support she received and felt she was able to be responsive and thoughtful in her interactions throughout the study, and felt that, with time, all groups developed a sense of bonding and increasing trust. Eventual conflicts and disagreements were allowed to surface and were not seen as problems but as a natural part of the interaction. However, she raised concerns about the possibility of missing important facial or gestural expressions, due to the online format. Professionally, she considered her participation to be a “transformative experience”, which helped her “listen more carefully, with curiosity and interest”, as well as learn ways to carry out research “in a more human and caring manner”. Finally, she emphasized the importance of a space for “imagination, art and fun”, during a period of social distancing and, for many, of suffering.

## Discussion

We conducted this study to determine the main effects of LuDiCa on behaviors relevant to social interaction in groups of autistic and neurotypical adolescents. Our intervention significantly increased the frequency of conversational acts among both groups of participants. Additionally, LuDiCa promoted more frequent initiation of conversation, sharing, and increased engagement in shared reading activities. The effect of LuDiCa on conversational acts was similar in both groups, suggesting that LuDiCa facilitates group interactions and two-way improvements in communication, rather than individual behaviors.

We also found that metacommunication acts remained stable or decreased throughout the procedure, likely due to increased adaptation to the virtual environment. Interestingly, we observed that the decrease in metacommunication acts was accompanied by an increase in dialogue around shared literary works. This specificity of the effect supports the functional relationship between LuDiCa and the frequency of dialogue among participants. Our study also demonstrated the importance of including the opinions of autistic adolescents in the assessment of social validity.

Our findings regarding initiation were balanced in groups A and B, but not in the other two groups, where there was a higher rate of initiation among neurotypical participants. Future research should investigate the roles of the facilitator and LuDiCa strategies in support of a more balanced. These results support the development of shared and dialogical reading protocols with autistic young people and future interventions in inclusive school settings.

LuDiCa facilitated dialogue around shared literary works and encouraged the emergence of sharing. This indicates its potential to create a safe environment that encourages the exchange of experiences, positively affecting double empathy. The decrease in sharing during maintenance suggests that our groups may have been influenced by the distancing questions used in the intervention.

Our approach to treating autistic and neurotypical adolescents as starting interaction on an equal footing and learning to interact together goes against the prevalent starting point of most peer social skills training studies, which, as we showed, tend to take it for granted that neurotypical students will be more socially skilled (as shown in their designation as “natural experts”). By creating opportunities for social interaction among adolescents without presupposing this unequal starting point, we were able to foster empathy and address conflicts and challenges in a neurodiverse context.

In future studies, we plan to deepen our understanding of how the depth of characters and emotional landscape of the work we used in this study contributed to results, for example, by comparing the effects of novels versus traditional tales, considering the greater depth of the characters in the latter, on instances of distancing and sharing. While employing a single-subject design focused on behavioral samples, we recognize the importance of incorporating more traditional randomized trials utilizing group statistics to bolster the evidence supporting the efficacy of LuDiCa.

## Conclusion

In conclusion, our study provides initial experimental evidence regarding the positive effects of LuDiCa on social interaction behaviors among autistic and neurotypical adolescents, in an inclusive setting. The findings support the efficacy of LuDiCa in promoting communication, fostering dialogue around shared literary works, and encouraging the emergence of sharing. These results underscore the potential of LuDiCa as a valuable tool for creating a safe and inclusive environment that facilitates meaningful exchanges.

Perhaps the most innovative aspect of this study was that we found a way, through the context of LuDiCa, of fostering interaction that successfully challenged the prevailing assumption that neurotypical adolescents should be regarded as “experts” in social skills. Unlike previous studies that assumed an inherent social advantage for neurotypical individuals, we intentionally approached all participants as equals, emphasizing the importance of interaction, mutual understanding, and sharing of thoughts and experiences. By rejecting the notion of an unequal starting point, we fostered an inclusive environment that promoted empathy and enabled meaningful social connections to develop. This unique aspect of our study highlights the significance of recognizing the value and potential of every individual, regardless of neurodiversity, in promoting positive social interactions.

### Supplementary Information

**Additional file 1.** Parent Interview Questionnaire

**Additional file 2.** Function and event analysis model

**Additional file 3.** Intervention fidelity assessment protocols

## Data Availability

The datasets and materials are available from the corresponding author on reasonable request.

## Abbreviations

APA: American Psychiatric Association
ASD: Autism spectrum disorder
BL: Baseline
LuDiCa: *Leitura Dialógica para Compreensão* (Dialogic Reading for Comprehension)
Mn: Maintenance
nd: Neurodivergent
nt: Neurotypical
PD: Pause for Dialogue,
PMI: Peer-mediated interventions
QDF: Questions directed to the facilitator
QDP: Questions directed to peers

## Footnotes

1 We opted for this nomenclature based on the guidelines of the American Psychological Association (2019), which guides people to be named according to their preference. Following the paradigm of neurodiversity, in which all forms of neurological diversity are valuable, and we must respect them as a natural form of human variation (den Houting, 2019), we will also use the term “neurodivergent” as the most general term for appointing autistic people with different support needs.

2 The Social Disability Movement emerged in the 1960s in the UK, by and for people with disabilities. The movement stresses the existence of social and physical barriers that impede justice and accessibility for all (Bampi et al. 2010).

3 The acronym was formed from the Portuguese - *Leitura Dialógica para a Compreensão.*

4 Hans Christian Andersen Award (*International Board on Books for Young People*, IBBY) for the whole work (1982); ALMA Award (*Astrid Lindgren Memorial Award*) by the whole of works (2004).

5 In fact, Bento had suggested this during sessions already, and in two sessions, he and Ana agreed to stay online a bit longer and talk about other topics of interest (e.g., films and *animes*).
