# Supplementary material for: Effects of dialogic reading for comprehension (LuDiCa) on the social interaction of autistic adolescents and their peers
Source: Psicol Reflex Crit. 2024 Feb 2;37:4. doi: 10.1186/s41155-023-00283-x (PMC10837403; doi:10.1186/s41155-023-00283-x)
Supplement: Supplementary file 1 — Additional file 1. Parent Interview Questionnaire [file 41155_2023_283_MOESM1_ESM.docx]

**Additional file 1: Parent Interview Questionnaire**

Identification:

Teens’ full name:

Teens’ date of birth:

Name of person responsible for answering the questionnaire:

Parent's date of birth:

Application date:

**Social consciousness**

1) Imagine that your son/daughter is with a friend who is very sad but does not say that he is. How does your son/daughter react?

2) Imagine your son/daughter is at a party. What is your son/daughter favorite food? Suppose he/she is hungry and has a plate of this food on the party table, looking delicious. But no one is getting served and it seems very clear that the food is not yet to be touched. How would your son/daughter react?

3) When there is a sensitive subject that no one in the group wants to touch on, does your son/daughter notice that the subject is embarrassing and avoid talking about it?

4) Does your son/daughter usually give gifts to other people spontaneously? (does not need to be purchased, could be a card, etc.)? Can you give me an example?

5) Does your son/daughter show concern and try to help when he/she sees a person in distress? For example, if someone is clearly in pain or feeling unwell, does your son/daughter ask the person how they are feeling? Looking to do something for the person?

**Social Cognition**

1) Has it ever happened to you that your son/daughter didn't quite understand a joke, or understood it in a different way? Does he/she like jokes? Could you give me an example?

2) Let's say your son/daughter is in a group of boys his/her age and they start to make fun of him (make fun of him/her) (1) will he/she notice soon? What is his/her general reaction in these situations?

3) Does your son/daughter understand irony? For example, if someone went on vacation to the beach and sends a photo in the best way, enjoying the vacation, but with a text like "Too boring, this here!" Do you think this might confuse your son/daughter? Does he use irony?

4) What subject does your son/daughter most like to talk about? Okay let's assume your son/daughter is talking about X (insert favorite). But the person he/she is talking to starts looking at the clock and trying to interrupt the conversation, clearly showing that he/she doesn't want to continue talking anymore. How does your son/daughter react?

5) Does your son/daughter know young children, 3, 4, 5 years old, for example? Who? Let's assume that your son/daughter is talking or playing with X (insert child's name or reference). Will he change the way he talks or plays? Can you give me an example?

**Social Communication**

1) Let's assume that your son/daughter is feeling sick, and you need to explain to the doctor what he is feeling. Can he/she communicate what he/she is feeling easily? Can you describe pains, sensations, or symptoms clearly?

2) Does your son/daughter use words like tired, sad, upset, happy, stressed, excited to talk about himself/herself? If so, could you give some examples of situations?

3) Let's assume you're at the mall and can't find a store where there's something that your son/daughter really wants and that you're going to give him/her as a gift. Does your son/daughter take the initiative to ask for directions? If not, how does he/she resolve the situation?

4) Has your son/daughter ever called somewhere where he/she had to interact with strangers (eg cell phone or cable company) to resolve a problem? How it was?

5) When he/she talks with colleagues, cousins, or friends of his/her age, how is the interaction? Do you think he/she talks more, the same or less than the others? Can you give me examples?

6) Does your son/daughter use facial expressions and gestures when talking? Would you say more, less, or equal to peers of the same age?

7) Would you say that, in general, your son/daughter, in general, smiles a lot, a little, like other people?

**Social motivation**

1) What is your son’s/daughter's favorite pastime? Between doing X and going to a party, which one will he/she choose?

2) Does your son/daughter like to receive praise? Can you give an example?

3) Does your son/daughter miss school friends on vacation? Have you ever expressed longing for a friend who was distant or with whom you had no contact?

4) Has your son/daughter ever complained about feeling lonely? Can you describe the situation?

5) Who are the people your son/daughter spends most time with? What does he/she do with them?

**Restricted interests and repetitive behaviors**

1) What does your son/daughter like to do most? Can you talk a little more about that?

2) How does he spend his day? What do you do after school?

3) If your son/daughter has a whole day free, what, for sure, will he/she do or ask to do?

4) What are your interests, what do you like to talk about?

5) Does your son/daughter have any special movements or sounds they like to do or use to calm down?

6) How does your son/daughter react when there is a very sudden change in routine or plans? Could you give me an example?
