# Supplementary material for: Effects of dialogic reading for comprehension (LuDiCa) on the social interaction of autistic adolescents and their peers
Source: Psicol Reflex Crit. 2024 Feb 2;37:4. doi: 10.1186/s41155-023-00283-x (PMC10837403; doi:10.1186/s41155-023-00283-x)
Supplement: Supplementary file 2 — Additional file 2. Function and event analysis model [file 41155_2023_283_MOESM2_ESM.docx]

Additional file 2: Function and event analysis model

| **Book:** The yellow bag (Lygia Bojunga)  Chapter 7 Analysis – Terrible Goes Away (p. 85 to 91) | | | |
| --- | --- | --- | --- |
| **FUNCTIONS** | | | |
| *Identified function* | *Examples of evidence of function* | *Distancing question* |  |
| 1. Secondary Function: Raquel wakes up scared to Afonso's screams | *“I woke up with Afonso terrified: - Raquel, the Terrible ran away!.” |  |  |
| 1. Secondary Function: Afonso blames the closure. | *“- Surely the closure opened.” |  |  |
| 1. Secondary Function: Raquel gets mad at the closure when she learns of Terrible's disappearance. | *”- You annoying! How do you let the Terrible get away?”  “But the closure is silly, until today he hasn't learned to speak anything. It just stays in that click-click and that’s it.” |  |  |
| 1. Secondary Function: Raquel feels sorry for the Zipper when she sees it scratched. | *”That's when I saw that he was all scratched up inside, poor thing. The Terrible One must have fought him and he had no choice but to open it.” |  |  |
| 1. Primary Function: Raquel and Afonso are worried about the message left by Terrible. | Q1 – How do you think Raquel and Afonso were feeling at that moment? Why?  * They wake up in fright, they don't find it terrible, they notice the scratched clasp  * They find a note: “I went to fight the fight I had to fight. To show that I can still win.” | Q1 – What would everyone here do in a situation like this? When receiving a message like this, what would you do? |  |
| 1. Secondary Function: Afonso is worried about the possibility of Raquel's family waking up. | *"What if people wake up and don't see you?" |  |  |
| 1. Secondary Function: Afonso insecure about the possibility of defeating his cousin, Terrible. | *"What if he didn't win?" |  |  |
| 1. Primary Function: Raquel was determined to find the Terrible One, or at least some clue to his whereabouts. | Q2 – What did Raquel do to convince Afonso to go after Terrible? How?  *"So let's go!"; *"It's early: there's time to go and come back before everyone wakes up."; *"Let's go at once, Afonso!"; *"It's no use thinking about it, it's best to go there and see." | Q2 – What do you think they can find the worst in “Praia das Pedras”? |  |
| 1. Secondary function: “Praia das Pedras” as an unpleasant and dangerous context; bad feeling. | *””always half empty”; “at night then it becomes deserted”; “They were very tough people”; “...a hell of a mess”; “...fight drawing.”; “There was blood in the drawing.” |  |  |
| 1. Secondary Function: Terrible's risk of life and Afonso's feeling of sadness and worry | *"there were some feathers on the floor" (which belonged to the Terrible); |  |  |
| 1. Secondary Function: Raquel gets sleepy because Umbrella talked to Afonso. | *" He talked so much that I even lay down to sleep." |  |  |
| 1. Primary Function: High chance of the Terrible being killed or missing. Strong feeling of sadness and fear of Afonso. | Q3 – And now? How was Afonso feeling about his cousin?  “And the face getting worse”; "...the crest collapsed, the head was down and the tail feathers that were always so lively, became so withered that it was pitiful." | Q3 – And you now? How do you feel to see Afonso in this situation? |  |
| **EVENTS** | | | |
| 1 – Afonso wakes up Raquel and reports the disappearance of Terrible. | |  |  |
| 2 – Raquel fights with the clasp, blaming him for the disappearance of Terrible. | |  |  |
| 3 – Clasp appears scratched due to fight with Terrible. | |  |  |
| 4 – Afonso finds a note from Terrible saying he had gone to the fight he had to fight. | |  |  |
| 5 – Raquel decides to go to Praia da Pedras very early in the morning after Terrível. | |  |  |
| 6 – At Praia das Pedras, drizzling, they find: a wheel in the sand, a line drawing a fight, blood in the drawing and Feathers of the Terrible on the ground. | |  |  |
| 7 – Raquel and Afonso hear the Umbrella's moan of “help” and realize that it has fallen behind the rocks. | |  |  |
| 8 – Guarda-Chuva talks for a long time with Afonso, who gets worse and worse as he listens. | |  |  |
| 9 – When the Umbrella stops talking, Afonso asks Raquel to put the Umbrella in her bag. | |  |  |
| 10 – Afonso says that Guarda-Chuva tried to prevent Terrible from participating in the fight and ended up being beaten. | |  |  |
| 11 – Raquel asks Afonso if the Umbrella managed to see the fight. | |  |  |
| 12 – Afonso replies that Crista de Ferro won the fight. | |  |  |
| 13 – Afonso says that the Terrible was taken away, while picking up his souvenir feathers. | |  |  |
| 14 – Raquel looks at the wheel and thinks about the differences in use by children and adults | |  |  |
| 15 – Raquel asks Afonso if it would be different if they hadn't sewn the thought with a strong thread. | |  |  |
| 16 – Afonso does not respond and walks away quickly. | |  |  |
