# Supplementary material for: Effects of dialogic reading for comprehension (LuDiCa) on the social interaction of autistic adolescents and their peers
Source: Psicol Reflex Crit. 2024 Feb 2;37:4. doi: 10.1186/s41155-023-00283-x (PMC10837403; doi:10.1186/s41155-023-00283-x)
Supplement: Supplementary file 3 — Additional file 3. Intervention fidelity assessment protocols [file 41155_2023_283_MOESM3_ESM.docx]

Additional file 3: Intervention fidelity assessment protocols

| **Procedures Evaluation Criteria**  **(BL and Maintenance)** | **Yes** | **No** |
| --- | --- | --- |
| Started dialog |  |  |
| Was responsive to occasional initiations without following up |  |  |
| Demonstrated affection/playfulness |  |  |
| Demonstrated theatricality and expression in reading |  |  |
| Raised hypotheses about the content of the story (Maintenance); and/or if any participant already knew the tale |  |  |
| Conducted follow-up breaks to verify that participants were following |  |  |
| At the end of the reading of the session, it raised the opinion of the participants about the passage read |  |  |

| **Procedures Evaluation Criteria (Intervention - LuDiCa)** | **Yes** | **No** |  |
| --- | --- | --- | --- |
|  |  |  |  |
| Demonstrated affection/playfulness |  |  |  |
| Demonstrated theatricality and expression in reading |  |  |  |
| Resumed memories of other sessions |  |  |  |
| At the end of the reading of the session, it raised the opinion of the participants about the passage read |  |  |  |
| Performed the three pauses for dialogue (with 2 questions: (1) understanding; and (2) distancing |  |  |  |
|  | **Always** | **Sometimes** | **Little** |
| Showed that she listened and reinforced differentially (was responsive) |  |  |  |
| Expanded and/or reformulated the question |  |  |  |
| Provided examples and continued reading |  |  |  |
